# Allostasis as a core feature of hierarchical gradients in the human brain

**DOI:** 10.1162/netn_a_00240

**Published:** 2022-10-01

**Authors:** Yuta Katsumi, Jordan E. Theriault, Karen S. Quigley, Lisa Feldman Barrett

**Affiliations:** Department of Neurology, Massachusetts General Hospital and Harvard Medical School, Boston, MA, USA; Department of Psychology, Northeastern University, Boston, MA, USA; Department of Psychiatry, Massachusetts General Hospital and Harvard Medical School, Boston, MA, USA; Athinoula A. Martinos Center for Biomedical Imaging, Massachusetts General Hospital and Harvard Medical School, Boston, MA, USA

**Keywords:** Predictive processing, Predictive coding, Active inference, Interoception, Functional networks, Energetics

## Abstract

This paper integrates emerging evidence from two broad streams of scientific literature into one common framework: (a) hierarchical gradients of functional connectivity that reflect the brain’s large-scale structural architecture (e.g., a lamination gradient in the cerebral cortex); and (b) approaches to predictive processing and one of its specific instantiations called *allostasis* (i.e., the predictive regulation of energetic resources in the service of coordinating the body’s internal systems). This synthesis begins to sketch a coherent, neurobiologically inspired framework suggesting that predictive energy regulation is at the core of human brain function, and by extension, psychological and behavioral phenomena, providing a shared vocabulary for theory building and knowledge accumulation.

## Author Summary

Allostasis refers to the process by which the brain anticipates the needs of the body and attempts to meet those needs before they arise, and is one specific instantiation of a broader predictive processing framework. In this perspective article, we propose that allostasis is a basic function of the human brain subserved by an intrinsic architecture composed of two hierarchical functional gradients. Our framework, based on a synthesis of multimodal and multiscale evidence across species, begins to sketch a coherent, neurobiologically inspired research program suggesting that predictive energy regulation is at the core of human brain function, and by extension, psychological and behavioral phenomena, providing a shared vocabulary for theory building and knowledge accumulation.

## INTRODUCTION

A growing consensus in neuroscience is that progress in understanding the nature of mind and behavior must begin by seriously considering the evolution and development of the human brain (e.g., Cisek, 2019, 2021). This perspective article extends hypotheses found in our earlier work suggesting that one core function of a brain is to efficiently coordinate and regulate the energetic requirements of its body, consistent with evidence from vertebrate brain evolution, embryological development, and signal processing (Gee, 2018; Sterling & Laughlin, 2015; Striedter & Northcutt, 2020). Specifically, we synthesize theoretical and empirical evidence emerging from two streams of literature: (a) hierarchical gradients of functional connectivity that reflect the brain’s large-scale structural architecture (for example, a lamination gradient in the cerebral cortex; e.g., Barbas, 2015; Hilgetag & Goulas, 2020; Margulies et al., 2016; Zhang et al., 2019), and (b) predictive regulation of the body’s energy resources, called *allostasis* (Schulkin & Sterling, 2019; Sennesh et al., 2021; Sterling, 2012; Sterling & Laughlin, 2015). Allostasis is the process by which the brain anticipates the needs of the body and attempts to meet those needs before they arise, and is one specific instantiation of a broader *predictive processing* framework that has developed to understand motor movements, perceptions, cognitions, emotions, and even consciousness (e.g., Clark, 2013, 2016; Denève & Jardri, 2016; Friston, 2010; Friston et al., 2017; Hohwy, 2013; Hohwy & Seth, 2020; Hutchinson & Barrett, 2019; Keller & Mrsic-Flogel, 2018; McNamee & Wolpert, 2019; Rao & Ballard, 1999; Seth, 2015).

Building on prior theorizing and evidence on allostasis from our group and others (Barrett, 2017; Barrett & Simmons, 2015; Hutchinson & Barrett, 2019; Khalsa et al., 2018; Kleckner et al., 2017; Owens et al., 2018; Petzschner et al., 2021; Petzschner et al., 2017; Pezzulo et al., 2015; Pezzulo et al., 2021; Schulkin & Sterling, 2019; Seth & Friston, 2016; Seth & Tsakiris, 2018; Stephan et al., 2016), as well as on papers dealing more generally with predictive processing in relation to bodily regulation or bodily sensing (e.g., Ainley et al., 2016; Allen, 2020; Allen & Friston, 2018; Allen et al., 2019; Seth, 2013; Seth et al., 2012; Smith et al., 2017), we propose that two of the large-scale functional gradients identified in multiple brain structures—in the cerebral cortex, including gradients in the isocortex and in the hippocampus (i.e., allocortex), as well as in the cerebellum—can be meaningfully interpreted as an intrinsic neural architecture that supports predictive processing, including allostasis. Our analysis extends the literature on bodily regulation by suggesting that allostasis is a *whole-brain* phenomenon, rather than attempting to localize it to a small set of brain regions. Our framework also extends the literature on hierarchical gradients by proposing that they may serve *domain-general* functions in the brain, offering an opportunity to understand how cognition, emotion, perception, and other psychological phenomena might emerge from a common set of computational ingredients. Identifying allostasis as a key element in the state space of a brain, which exists as a complex, nonlinear dynamical system that continually interacts with its body and the surrounding world, offers new opportunities to build a unified science of brain, body, and mind.

## THE BRAIN’S INTRINSIC SYSTEM FOR ALLOSTASIS

Evolutionary, developmental, and anatomical studies of the vertebrate brain all suggest that its fundamental job is to efficiently regulate the body’s internal systems as an animal navigates its environmental niche. Predictive regulation is an improvement over reaction because reactive systems adapt only in the face of error, but any mistake is potentially fatal (Sterling, 2012; Sterling & Laughlin, 2015). Prediction also limits the extent to which incoming signals need to be encoded (Shannon & Weaver, 1949/1964), which may save the metabolic costs of learning predictable information (Sengupta et al., 2013; Theriault, Shaffer, et al., 2021; Theriault, Young, et al., 2021). Across the expanse of time, vertebrates evolved larger bodies, making new biological systems necessary (Gee, 2018; Striedter & Northcutt, 2020). These include systems for waste disposal, nutrient dispersal (e.g., renal system, respiratory system, cardiovascular system), as well as systems to sense their expanding niche (e.g., vision, audition, olfaction). As biological systems proliferated, so did the need grow for a brain to actively coordinate and regulate them. Accordingly, rudimentary neurons in a ganglion that sat atop the spinal cord became brain stem structures (e.g., the optic tectum, or the superior colliculi in mammals; the ventral hypothalamus), and eventually, novel structures such as the diencephalon and the telencephalon (including the cerebral cortex) emerged (Gee, 2018; Striedter & Northcutt, 2020). Embryological development is consistent with this evolutionary narrative, in that the brain, visceral systems, and exteroceptive systems all arise from adjacent locations on the neural plate (Feinberg & Mallatt, 2013; Nieuwenhuys & Puelles, 2016). This narrative, and the embryological evidence, suggests that the regulation of the body is a core consideration for brain evolution, brain development, and brain function in organisms.

The viability of this hypothesis is bolstered by tract-tracing and cytoarchitectonic evidence from mammalian brains (e.g., Barbas, 2015; Carmichael & Price, 1996; Evrard et al., 2014; Mufson & Mesulam, 1982; Öngür et al., 2003; Vogt & Pandya, 1987; Vogt et al., 1987). We summarized these findings in a recent paper showing their congruence with functional connectivity of the human brain “at rest,” while the brain is not being probed by exogenous stimuli (e.g., in an experimental task) but is still attached to body and regulating its internal systems (Kleckner et al., 2017). In primates and other mammals, cortical regions known as *limbic* cortices form a ring around the thalamus, hypothalamus, and some brainstem regions (Willis, 1664) on the medial wall of each hemisphere continuing into temporal and orbitofrontal cortices (Barbas, 2015; Chanes & Barrett, 2016). Limbic cortices send monosynaptic projections to subcortical (e.g., hypothalamus) and brainstem (e.g., periaqueductal gray, parabrachial nucleus, nucleus of the solitary tract) structures that regulate the internal systems of the body, including the autonomic nervous system, immune system, and endocrine system. Limbic cortices traditionally refer to the hippocampus, amygdala, olfactory cortex, and the most medial portions of the cingulate cortices (and in some papers, portions of the basal ganglia). In some nomenclatures (e.g., Barbas, 2015), the “limbic” designation also includes areas that elsewhere are referred to *paralimbic* regions of the brain, which are zones of cytoarchitectural transition between allocortical tissue and more prototypic isocortex, such as ventral anterior insula/posterior orbitofrontal cortex, temporal pole, cingulate cortices, and entorhinal cortex (see Mesulam, 2000). In this paper, we follow Barbas (2015) and refer to the entire ensemble as “limbic” (for a history of the “limbic” concept in neuroanatomy, see Lautin, 2001; Roxo et al., 2011).

**Figure 1.**
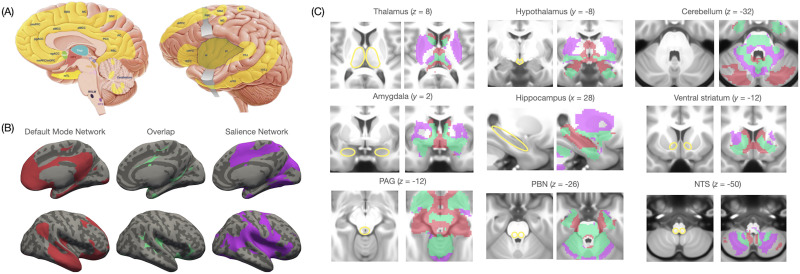
A schematic summary of findings from Kleckner et al. (2017) illustrating the intrinsic neural system for allostasis. (A) Yellow highlights indicate areas of the cerebral cortex corresponding to the intrinsic allostatic system. (B) The intrinsic allostatic system consists of two overlapping intrinsic functional networks, which are conventionally called the “default mode network” and the “salience network.” (C) Hippocampal and subcortical connectivity of the two functional networks constituting the allostatic system; colors correspond to those used in panel B; x, y, and z values correspond to the MNI coordinates in millimeters. Panel A was modified from the figure originally published in https://dana.org/article/interoception-the-secret-ingredient/.

We identified homologous locations of these limbic cortices in the human brain based on the coordinates from previous neuroimaging studies. Using functional magnetic resonance imaging (fMRI) data collected at rest in more than 500 participants (split into discovery and replication samples), we computed whole-brain functional connectivity maps for each cortical limbic region, estimated as correlations in time courses of low-frequency blood oxygen level–dependent (BOLD) signals for the voxels in each region with the voxels in the rest of the brain (Kleckner et al., 2017). An unsupervised clustering analysis of both discovery and replication maps revealed two spatially overlapping ensembles that are commonly referred to as the “default mode network” and the “salience network,” which constitute the brain’s intrinsic system for allostasis (Figure 1).

The interpretation of the default mode and salience networks and their overlap as an intrinsic allostatic neural system is consistent with connectivity profiles of the two networks. The cortical limbic nodes in both networks have extensive functional connectivity with subcortical and brainstem structures that are thought to be important for allostasis (Figure 1C). These limbic nodes also exhibit connectivity with the cerebellum and the hippocampus, consistent with tract-tracing evidence identifying their connections to the structures that process outgoing visceromotor and incoming interoceptive signals (Pisano et al., 2021; Suarez et al., 2018; Vertes, 2015; Zhu & Wang, 2008). Functional imaging evidence also implicates activity in the default mode and salience networks with bodily regulation. For instance, cardiac activity at rest correlates with low-frequency BOLD signal fluctuations in these networks (Valenza et al., 2019), and tasks examining autonomic regulation of the cardiovascular system consistently elicit activation within their cortical nodes and subcortical extents (Beissner et al., 2013; Gianaros & Wager, 2015).

There are several notable observations that can be drawn from the intrinsic allostatic system that provide opportunities for novel discoveries in the growing field of allostatic regulation. First, as the default mode and salience networks maintain allostasis, they play a role in modeling the interoceptive consequences of allostatic regulation. Recent work suggests that successful allostasis may require the predictive construction of interoceptive signals (anticipation of physiological changes in the body due to some future behavior; Barrett, 2017; Gu & FitzGerald, 2014; Pezzulo et al., 2015). Regions in the default mode and salience networks have been discussed in computational models of allostasis and other forms of bodily regulation as well as interoception (Ainley et al., 2016; Allen, 2020; Allen et al., 2019; Barrett & Simmons, 2015; Khalsa et al., 2018; Owens et al., 2018; Petzschner et al., 2017; Pezzulo et al., 2015; Seth, 2013; Seth & Friston, 2016; Seth et al., 2012; Smith et al., 2017; Stephan et al., 2016). These models propose that visceromotor regions (e.g., anterior insula, anterior midcingulate cortex, and subgenual anterior cingulate cortex) are broadly involved in issuing signals to infer the causes of interoceptive signals and predict their trajectories into the future. Some of these models consider regions that are part of the default mode network to be higher in the processing hierarchy than regions of the salience network in the brain. Specifically, the former is hypothesized to infer the meaning of interoceptive signals based on past experience (Smith et al., 2017) or issue predictions to visceromotor regions within the salience network based on the brain’s beliefs about its capacity to successfully perform allostasis (i.e., “metacognition”; Stephan et al., 2016). These views are overall consistent with our framework in which agranular areas (i.e., an isocortical region with neurons that configure into a relatively undifferentiated superficial layer [corresponding to layers II and III] and lacking a fully differentiated layer IV) in the default mode network are thought to be crucial for the initiation of prediction signals that help give meaning to incoming sensations by generalizing from similar past experiences, which we have described as a process of continual ad hoc category construction (see **Prediction as a domain-general computational process in the brain**, below). The conceptual categories that are constructed as prediction signals constitute the brain’s internal model of its body in the world (Barrett, 2017; Kleckner et al., 2017).

Second, modeling of the sensory consequences of allostatic regulation also means modeling the expected changes in exteroceptive signals. That is, the brain does not detect signals in the world, but it models the features of those signals as they are transduced by the sensory surfaces of the body. Some of these features are high-dimensional (closer in detail to the signals from the sensory surfaces) and some are lower dimensional, compressed summaries (abstract features) such as affective valence and arousal or other “psychological” features. Further, to the extent that interoceptive signals (as modeled by the brain) act as control signals for allostasis (Sennesh et al., 2021), those signals may also play a role in the sampling of unanticipated exteroceptive signals (i.e., exteroceptive prediction errors). This hypothesis is supported by evidence that sampling of visual, auditory, and other data from the sensory surfaces of the body is statistically related to bodily signals such as heartbeats and respiration (e.g., Al et al., 2020; Aspell et al., 2013; Galvez-Pol et al., 2020; Grund et al., 2022; Kluger et al., 2021; Kunzendorf et al., 2019; Zelano et al., 2016).

Third, and relatedly, these findings suggest that allostasis may play a role in learning (i.e., processing of prediction errors). Dorsal mid to posterior insula, which functions as primary interoceptive cortex (Avery et al., 2015; Nieuwenhuys, 2012), is a point of overlap for the default mode and salience networks (Figure 1B), suggesting that processing of unanticipated interoceptive and exteroceptive signals (i.e., prediction errors) may be influenced by their predicted allostatic relevance. This hypothesis is supported by observations that limbic cortices in both networks show connectivity with brainstem nuclei that give rise to the neuromodulators involved in attention and neural excitability (e.g., ventral tegmental area, substantia nigra, dorsal raphe nucleus, and locus coeruleus; Bär et al., 2016; Price & Drevets, 2010). These findings suggest that attention and levels of consciousness may also be influenced by allostatic regulation.

Fourth, further extending this hypothesis, our view is that allostasis is always operating constantly regardless of whether an animal is awake (active) or at rest. A brain must always coordinate internal bodily systems and anticipate upcoming energy needs (Schulkin & Sterling, 2019), although at rest, those needs differ from the energic needs during active periods. For example, in contrast to active periods, digestion, waste secretion, and immune functions continue during rest (either asleep or quiescent). Further, the primary source of metabolic fuel also changes from active to resting periods (i.e., a shift from greater carbohydrate utilization to greater lipid utilization, respectively), and during quiescent/dark periods, physiological processes such as genomic replication that are especially sensitive to disruption by UV light are prioritized (Asher & Schibler, 2011; Gerhart-Hines & Lazar, 2015). It has been suggested that circadian cycling provides an important means by which allostatic (predictive) regulation of metabolism can occur (Asher & Schibler, 2011), and there is an intimate and evolutionarily long-standing coordination of circadian and metabolic control across tissues (e.g., cardiac muscle, skeletal muscle, gut microbiome; reviewed in Gerhart-Hines & Lazar, 2015) to mobilize resources to the body’s internal systems where they would be needed the most (Schulkin & Sterling, 2019). In addition, when the brain is perturbed with fewer exteroceptive prediction errors (e.g., in sleep), the intrinsic allostatic system may help refine the brain’s internal model of its body in the world, for instance, by removing redundancies in the model and thus reducing its complexity or generating “fictive” prediction error signals to train the model (for similar arguments, see Barron et al., 2020; Pezzulo et al., 2021).

Finally, the allostatic system may play a pivotal role in signal integration in the brain more generally. The default mode and salience networks overlap in and contain the highest proportion of “rich-club” hubs, defined as regions showing the densest anatomical connections within the cerebral cortex. These rich-club hubs are interpreted as the brain’s backbone for their central role in neural communication and synchrony (van den Heuvel & Sporns, 2011, 2013). Rich-club hubs that are limbic in structure (vs. non-limbic) exhibit topological properties more suited to function as “high-level” connectors, integrating already highly integrated information across modules or communities of regions (Zhang et al., 2020). Taken together, these findings support the existence of an intrinsic allostatic system in humans that is anatomically central and contributes to information integration and coordination in the entire brain as well as between the brain and the body.

## PREDICTION AS A DOMAIN-GENERAL COMPUTATIONAL PROCESS IN THE BRAIN

That allostasis is one of the brain’s core tasks is further supported by converging evidence for predictive processing models about bodily regulation and/or interoception. A variety of specific proposals abound (Ainley et al., 2016; Allen & Friston, 2018; Allen et al., 2019; Hohwy & Seth, 2020; Hutchinson & Barrett, 2019; Khalsa et al., 2018; Owens et al., 2018; Parr et al., 2018; Petzschner et al., 2021; Petzschner et al., 2017; Pezzulo et al., 2015; Pezzulo et al., 2021; Schulkin & Sterling, 2019; Seth, 2013; Seth & Friston, 2016; Seth et al., 2012; Seth & Tsakiris, 2018; Smith et al., 2021; Smith et al., 2017; Stephan et al., 2016), but they are united by three components that are thought to be implemented in a hierarchical arrangement in the brain’s architecture: (a) *prediction signals* that the brain generatively constructs using memory—or alternatively, an “internal model” (e.g., Berkes et al., 2011), “top-down” processing (e.g., Friston, 2010; Jordan & Keller, 2020; Rao & Ballard, 1999), a “forward model” (e.g., Wolpert et al., 1998), or “feedback” signals (e.g., Lamme & Roelfsema, 2000); (b) *prediction errors* (or “bottom-up” processing, or “feedforward” signals) that encode the differences between predicted sensory inputs and incoming sense data from the body’s sensory surfaces; and (c) *precision signals* (or attention signals or executive control) that modulate the strength and durability of predictions and prediction errors, and their ability to access motor control and influence behavior (Feldman & Friston, 2010; Kanai et al., 2015). Prediction errors are potential teaching signals, but their capacity to update predictions is thought to depend on how they are weighted by precision signals, which are interpreted as the predicted value of the allostatic information they will provide, or “salience” (Barrett, 2017; see also Parr & Friston, 2019, for a discussion of salience). Prediction signals are also thought to be weighted by their estimated value to explain the incoming sense data, which may correspond to their estimated prior probabilities (Barrett, 2017; Feldman & Friston, 2010; Kanai et al., 2015). The advantage of predictive processing for an organism is that learning accumulates to build an internal model of its body in the world, from which top-down predictions can be made and allostasis can be achieved.

**Figure 2.**
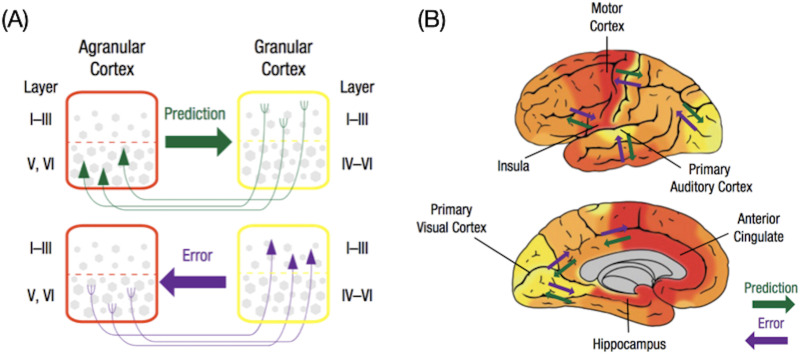
Hypothesized flow of prediction and prediction error signals in the human brain. (A) Information flow between cortical regions is predicted by the relative degree of laminar development in the communicating columns. Prediction signals flow from deep layers of less granular cortices (e.g., agranular areas with undifferentiated layers II and III and no layer IV) and terminate in superficial layers of more granular cortices (e.g., dysgranular/granular cortices, where layers II and III are differentiated and layer IV is rudimentary [dysgranular] or well-defined [granular]). Prediction error signals flow in the opposite direction, from superficial layers of more granular cortices to deep layers of less granular cortices (Barbas, 2015; García-Cabezas et al., 2019; as discussed in Barrett, 2017; Hutchinson & Barrett, 2019). (B) This cytoarchitectural gradient is thought to support lossy information compression in the cerebral cortex. That is, sensory prediction errors ascend the cortical hierarchy from superficial layers of (granular) sensory regions, first flowing to (less granular) heteromodal and motor cortex (Barbas & García-Cabezas, 2015) to dysgranular/agranular limbic regions involved in visceromotor control. As sensory prediction errors ascend along this laminar gradient, high-dimensional sensory signals are compressed and reduced in dimensionality (Barrett, 2017; Finlay & Uchiyama, 2015; for a related view, see Bastos et al., 2020) and become multimodal summaries (e.g., Braga et al., 2013; Sepulcre et al., 2012; Szinte & Knapen, 2020). The signals in limbic regions, then, may implement some of the most compressed, multimodal representations in the cerebral cortex that guide the formation of prediction signals. Prediction signals may ultimately control action and construct perception and subjective experience (Allen & Friston, 2018; Barrett, 2017; Fotopoulou & Tsakiris, 2017; Khalsa et al., 2018; Owens et al., 2018; Petzschner et al., 2017; Sennesh et al., 2021; Seth & Friston, 2016; Seth & Tsakiris, 2018; Stephan et al., 2016). Figure adapted from Hutchinson and Barrett (2019), with permission.

It has been proposed that structural properties of the cerebral cortex support the flow of prediction and prediction error signals. In macaque monkeys and other mammals, over 30 years of tract-tracing evidence demonstrates a cytoarchitectural substrate for this flow of signals, where predictions flow according to granular development (Figure 2; Barbas, 2015; García-Cabezas et al., 2019). Granular (or eulaminate) cortices have six definable cell layers, dysgranular areas have only a rudimentary layer IV, and agranular areas lack it altogether (Barbas, 2015; García-Cabezas et al., 2019). In addition, the size and connectivity of pyramidal neurons are different, with granular cortices made up of many small neurons with few connections, whereas agranular cortices contain fewer, larger neurons with many more connections (for a discussion, see Finlay & Uchiyama, 2015). The central observation is that limbic cortices are either agranular or dysgranular in laminar organization, and are thought to function as the most powerful feedback (i.e., prediction) regions in the brain (Joyce & Barbas, 2018). That is, prediction signals stem from the same neurons as those that are responsible for allostatic regulation of the body (Figure 1).

In prior work (Barrett, 2017; Barrett & Simmons, 2015; Chanes & Barrett, 2016), we hypothesized that prediction signals begin as visceromotor control signals in agranular and dysgranular limbic cortices, all of which are located in the default mode and salience networks of the intrinsic allostatic system (e.g., subgenual anterior cingulate cortex, entorhinal cortex, anterior midcingulate cortex, ventral anterior insula/posterior orbitofrontal cortex). Prediction signals descend from the deep layers of these cortices to subcortical and brain stem nuclei, which proceed to the spinal cord to coordinate and regulate the body’s internal systems. At the same time, collateral axons carry efferent copies of visceromotor predictions both to primary motor cortex (as skeletomotor prediction signals) and to primary interoceptive, visual, auditory, and somatosensory cortices as sensory prediction signals, either directly or via polysynaptic connections (for similar views of skeletomotor efferent signals, see Adams et al., 2013; Bastos et al., 2012). Primary motor cortex has a definable layer IV, but it is less well developed than most primary sensory areas (Barbas & García-Cabezas, 2015), meaning that primary motor cortex is hypothesized to send sensory prediction signals (Barrett, 2017). The same is true of primary interoceptive cortex, which has less laminar differentiation and likely sends sensory prediction signals to primary visual, auditory, and somatosensory cortices (Barrett, 2017; Chanes & Barrett, 2016). The exceptions to this hypothesis are primary olfactory and gustatory cortices, which are dysgranular in laminar organization, and therefore are at the same level of the predictive hierarchy as the visceromotor regions issuing allostatic control signals.

In this view, behavior and mental events involve a coordination of predictions: visceromotor predictions that regulate the internal milieu to make energetic resources available for skeleotomotor movements and experience, skeletomotor prediction signals that prepare the body for movement, interoceptive prediction signals related to affective features (i.e., low-dimensional representation of interoceptive sensations; Barrett & Bliss-Moreau, 2009), and exteroceptive sensory prediction signals that prepare the sensory surfaces of the body to receive upcoming sensory signals. Incoming interoceptive and exteroceptive signals either confirm or constrain these predictions. Interoceptively, ascending viscerosensory signals are carried along the vagus nerve and small diameter C and Aδ fibers (Craig, 2002), via various thalamic nuclei, and salient, unanticipated sensory inputs will be encoded as interoceptive prediction errors (e.g., reward or aversive prediction errors; Seth et al., 2012). Salient exteroceptive signals arriving from the sensory surfaces are also encoded as prediction errors, which, together with interoceptive prediction errors, modify the internal model and future predictions that constitute skeletomotor and visceromotor action plans, optimized to deal with particular sensory events. Both prediction and prediction error signals are subject to modulation by precision signals. This suggests that, from birth to death, the intrinsic allostatic system may be sending a cascade of reference signals to the body and predict the sensory consequences of those reference signals as sensory prediction errors that ascend the hierarchical arrangement of neurons along the cerebral cortex.

## HIERARCHICAL GRADIENTS IN THE CEREBRAL CORTEX AND THEIR ROLE IN PREDICTIVE PROCESSING

The hypotheses discussed so far propose that signal propagation in predictive processing is coordinated along a cytoarchitectural gradient in the cerebral cortex. Recent neuroimaging research examining intrinsic functional connectivity in the human brain suggests that the cerebral cortex is functionally organized along *multiple* gradients (Bethlehem et al., 2020; Margulies et al., 2016; Paquola et al., 2020; Paquola et al., 2019; Shafiei et al., 2020). In a recent series of studies, we have proposed that two of these gradients represent components of predictive processing (Katsumi et al., 2021; Zhang et al., 2019).

**Figure 3.**
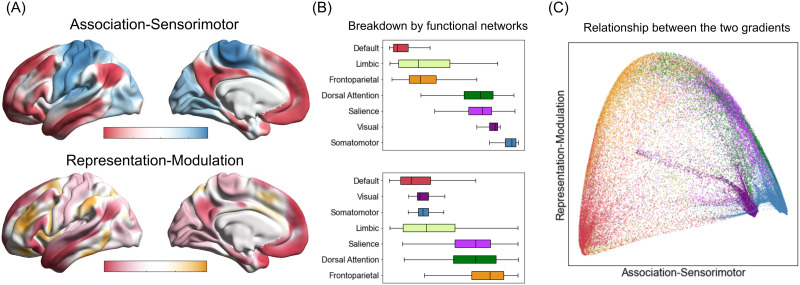
Two of the dominant functional connectivity gradients identified in the cerebral cortex. (A) For each connectivity gradient, the proximity of colors can be interpreted as greater similarity of connectivity patterns between a pair of cortical vertices. (B) Boxplots show medians and interquartile ranges of gradient values in canonical functional networks (Yeo et al., 2011), ordered by median values. Conventional network labels are used here, but note that “default” and “limbic” networks are not always distinguished in the literature (e.g., Kong et al., 2019) and both contain agranular, limbic tissue (Kleckner et al., 2017). (C) A scatterplot illustration of the two connectivity gradients. Colors correspond to those used in panel B to depict functional networks.

Typically, functional connectivity gradients are computed by constructing an affinity matrix, which describes the similarity of connectivity profiles in a set of data points (e.g., voxels, surface vertices, or parcels). A dimensionality reduction technique is then used to decompose this matrix into principal eigenvectors describing axes of largest variance (for additional technical details, see Vos de Wael et al., 2020). Each eigenvector can be used to identify gradual transitions in the pattern of functional connectivity within a given structure, thus yielding a connectivity “gradient.” We have recently proposed that two of the dominant and commonly identified connectivity gradients in the cerebral cortex are consistent with the role of cortical ensembles in predictive processing (as hypothesized in Barrett, 2017; Figure 3), which are discussed in detail below.

The principal gradient (explaining the largest amount of variance in connectivity profiles) is anchored at one end by the default mode and “frontoparietal” networks, and at the other end by the exteroceptive sensory and motor (e.g., somatomotor, visual) as well as salience networks. Here, we refer to this gradient as the *association-sensorimotor* gradient, although it has also been called a “transmodal-unimodal” gradient (e.g., Margulies et al., 2016). Converging evidence from neuroimaging, histological, transcriptomic, and electrophysiological studies identifies this gradient as a dominant axis of feature organization in the cerebral cortex, highlighting its role as a domain-general organizational motif (reviewed in Sydnor et al., 2021). We propose that this gradient can be interpreted through the lens of predictive processing, such that it separates regions involved in representing prediction signals on one end, and prediction error signals on the other.

At the “association” end, the default mode network is thought to construct highly compressed multimodal representations, which enable the initiation of prediction signals that constitute the brain’s internal model of its body in the world, guiding motor actions and making perception possible (Barrett, 2017; Buckner, 2012; Smith et al., 2017; Stawarczyk et al., 2021). Topographical features unique to the default mode network may facilitate these computations: Regions of the default mode network are, in certain cases, multiple synapses from primary exteroceptive sensory areas, allowing ample opportunity for multimodal compression—that is, the construction of abstract features (for a similar view, see Chanes & Barrett, 2016; Margulies et al., 2016; Smallwood et al., 2021). This is consistent with the role of this network in conceptual processing (Fernandino et al., 2016) and ad hoc category construction that gives meaning to sensory inputs (as discussed in Barrett, 2017).

The frontoparietal network is thought to have two major roles: (a) estimating the precision of prediction signals by suppressing predictions whose priors are very low, and when necessary, (b) sculpting and maintaining predictions longer than the several hundred milliseconds it takes to process imminent prediction errors (Barrett, 2017; see Smith et al., 2017, for a similar view). This is consistent with other accounts of the frontoparietal network’s function that its subnetwork acts as an extension of the default mode network and regulates complex introspective processes (Dixon et al., 2018). This may also explain why the default mode and frontoparietal networks exhibit longer timescales of cortical processing than any other functional network in the cerebral cortex (Hasson et al., 2015; Raut et al., 2020).

At the “sensorimotor” end of the association-sensorimotor gradient, exteroceptive sensory networks process sensory inputs that continually confirm or refine predictions made from the brain’s internal model of its body in the world. The salience network is hypothesized to estimate the precision of prediction error signals by altering the gain on prediction error signals as they propagate from the sensory periphery, reflecting confidence in the reliability and quality of incoming sensory information and its predicted relevance for allostasis. The salience network, then, may help the brain adjust its internal model to the energetic conditions of the body (Barrett, 2017). This proposal is consistent with accounts of salience network function in both attention regulation (Power et al., 2011; Touroutoglou et al., 2012; Uddin, 2015; Ullsperger et al., 2014) and multisensory integration (Craig, 2009; Sepulcre et al., 2012), and with computational models of interoception that highlight the role of salience network regions in estimating the precision of ascending interoceptive prediction error signals (e.g., Ainley et al., 2016; Allen, 2020; Allen et al., 2019; Seth et al., 2012). Our interpretation of the association-sensorimotor gradient provides an opportunity to integrate evidence from multimodal and multiscale approaches into a common framework. Specifically, this functional gradient is consistent with a cytoarchitectural gradient discussed above, which is hypothesized to support the flow of prediction and prediction error signals (Figure 2). It is also overall consistent with findings from recent studies investigating cortical myeloarchitecture, which identified a similar principal gradient spanning primary sensory and limbic regions (Huntenburg et al., 2017; Paquola et al., 2019).

The second gradient consistently identified in the literature is anchored at one end by the default mode and exteroceptive sensory networks and at the other end by the salience and frontoparietal networks. We refer to this gradient as a *representation-modulation* gradient, separating ensembles involved in the representation of low-dimensional multimodal summaries of brain states or more precise sensorimotor signals (Fernandino et al., 2016) from those involved in modulating these representations (e.g., via attention regulation, goal maintenance, strategy selection, or performance monitoring; Corbetta & Shulman, 2002; Dosenbach et al., 2007; Miller & Cohen, 2001; Uddin, 2015). This gradient has also been described as a “multiple demand” gradient (Genon et al., 2021; Paquola et al., 2020; Valk et al., 2021), as modulatory networks are often engaged in the face of task-based cognitive demands (Assem et al., 2020; Duncan, 2010; Fedorenko et al., 2013). Interpreted in terms of predictive processing, this gradient distinguishes regions that represent prediction and prediction error signals from regions that implement attentional modulation to compute the precision of these signals.

Another gradient commonly reported in studies of functional connectivity gradients is anchored at one end by the visual network and at the other end by the somatomotor network (Bethlehem et al., 2020; Margulies et al., 2016; Mckeown et al., 2020), suggesting a segregation of exteroceptive sensory systems. The anatomical and multiscale features of this gradient are not yet clear and the functional implications of this segregation remain unknown. The role of this gradient in predictive processing, therefore, awaits further evidence about its anatomical and functional features. Current evidence shows that this gradient appears dominant in newborn infant brains, explaining the largest magnitude of variance in intrinsic functional connectivity (Larivière et al., 2020). This gradient seems to remain dominant until the transition to adolescence, at which point the association-sensorimotor gradient supersedes (Dong et al., 2021). It is unclear what this means, however, given that both the visual and the somatomotor networks are still in development during infancy and make substantial advances in neurotypical development within the first year of life (Hadders-Algra, 2018; Johnson, 2013). Integration of the visual-somatomotor gradient with the current predictive processing framework is an opportunity for future research.

The relevance of the association-sensorimotor and representation-modulation gradients for allostasis is further substantiated by multimodal evidence identifying similar gradients within specific regions of the cerebral cortex. Specifically, existing computational models of bodily regulation (including allostasis) and interoception highlight the contribution of a functional gradient within the insular cortex, such that the posterior and mid insula represents interoceptive (and exteroceptive) information, whereas the anterior insula integrates multimodal information and exerts visceromotor control (Allen et al., 2019; Allen & Friston, 2018; Barrett & Simmons, 2015; Smith et al., 2017; Stephan et al., 2016). Extending this view, a recent study examining myeloarchitectural gradients within the entire insular cortex showed that the principal gradient captures gradual transitions from the posterior to anterior insula; this gradient corresponded with a shift in functional connectivity profiles from primarily sensorimotor to greater affiliation with the salience network (Royer et al., 2020). This is consistent with the cytoarchitectural gradient from granular (posterior) to agranular (anterior) insula, corresponding to a hypothesized flow of prediction and prediction error signaling within this cortical area (as discussed in Barrett & Simmons, 2015). This posterior–anterior insular gradient, then, appears to correspond to the cortical association-sensorimotor gradient, with posterior insula at the sensorimotor end, and the anterior insula at the association end. Additionally, the second most dominant myeloarchitectural gradient in the insula showed transitions from the posterior and ventral subregions to the dorsal anterior insula; this gradient corresponded with a shift in functional connectivity patterns from uniquely sensorimotor to uniquely modulatory/attentional (i.e., salience, dorsal attention, and frontoparietal networks; Royer et al., 2020), suggesting the correspondence of this insular cytoarchitectural gradient with the cortical representation-modulation gradient. This evidence is consistent with the hypothesis that the precision of ascending sensory prediction errors is computed along intra-insular gradients (Ainley et al., 2016; Allen, 2020; Allen et al., 2019) or more generally by the salience network (as discussed in Barrett, 2017).

## ROLE OF HIERARCHICAL HIPPOCAMPAL AND CEREBELLAR GRADIENTS IN PREDICTIVE PROCESSING

**Figure 4.**
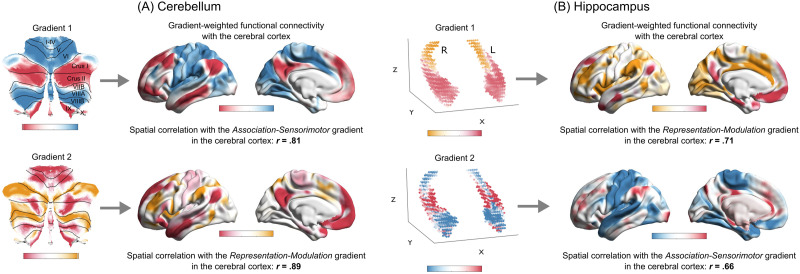
Functional connectivity gradients of the cerebellum and the hippocampus. Gradient-weighted functional connectivity maps represent, for a given gradient, the relationship between a given pair of structures in terms of their functional connectivity profiles. For example, voxels in Crus I and Crus II of the cerebellum anchoring one end of its Gradient 1 (depicted in red in a flat map above) showed relatively greater (positive) functional connectivity with the default mode network in the isocortex than did cerebellar voxels anchoring the other end of the same gradient.

Beyond the isocortex, the association-sensorimotor and representation-modulation gradients also appear to govern the functional organization of other structures in the brain, such as the cerebellum (Guell, Schmahmann, et al., 2018) and the hippocampus (i.e., allocortex; Vos de Wael et al., 2018). We built on these prior studies to assess the extent to which the functional connectivity gradients in these structures corresponded with the two isocortical gradients (Katsumi et al., 2021). In the cerebellum, the principal gradient captured a bilateral dissociation of lobules IV, V, and VI and lobule VIII from the posterior part of Crus I and II and the medial part of lobule IX, whereas the second dominant gradient distinguished bilaterally the anterior parts of Crus I and Crus II along with lobule VIIb from the rest of the cerebellar cortex (Figure 4A). In the hippocampus, the principal gradient revealed a functional dissociation along the longitudinal axis, whereas the second most dominant gradient additionally captured variation along the transverse (i.e., medial-lateral) axis (Figure 4B).

To characterize these cerebellar and hippocampal connectivity gradients in terms of their relations to the isocortex, we calculated intrinsic functional connectivity maps for each of these isocortical structures weighted as a factor of voxel-wise gradient values. For example, to characterize how a given cerebellar gradient related to the isocortex, we computed a cerebello-isocortical connectivity map for each cerebellar voxel and multiplied it by the corresponding gradient value for that particular voxel. In this way, the pattern of functional connectivity between each cerebellar voxel and all isocortical vertices was weighted by the position of the voxel on the cerebellar gradient. These voxel-wise, gradient-weighted cerebello-isocortical connectivity values were summed over all cerebellar voxels, resulting in a single isocortical projection of the cerebellar gradient. We repeated this procedure for each gradient derived for the cerebellum and the hippocampus. This procedure allowed us to project cerebellar and hippocampal gradients onto the isocortex, thus revealing the extent to which they spatially corresponded with the isocortical association-sensorimotor and representation-modulation gradients. We found that the principal cerebellar gradient strongly corresponded with the association-sensorimotor gradient, whereas its second most dominant gradient corresponded with the representation-modulation gradient. In contrast, in the hippocampus, the principal gradient strongly corresponded with the representation-modulation gradient, whereas its second most dominant gradient corresponded with the association-sensorimotor gradient (see cortical surface maps in each panel, Figure 4).

The observed correspondence between connectivity gradients in the isocortex, the cerebellum, and the hippocampus suggests a starting point for developing one unified, integrative view of brain function, where allostatic regulation may be one of the core computational features. This view extends earlier research examining computational capacities of these structures, which have been described in terms of predictive processing (Barron et al., 2020; Ito, 2008; Liu et al., 2018; Pezzulo et al., 2017; Wolpert et al., 1998). Converging empirical evidence supports the hypothesis that both the cerebellum and the hippocampus are involved in allostasis and interoception. The cerebellum exhibits direct or indirect anatomical connections with various subcortical structures implicated in allostasis, including the hypothalamus, periaqueductal gray, nucleus of solitary tract, and amygdala (Zhu & Wang, 2008). Of note, direct and bidirectional connections between the cerebellum and the hypothalamus are thought to be critical for the regulation of the body’s internal systems, including cardiovascular, respiratory, gastrointestinal, and immune systems (Zhu et al., 2006). The hippocampus also receives rich inputs from subcortical structures, including the medial septum, amygdala, anterior thalamic nuclei, supramammillary nucleus of the hypothalamus, and brain stem nuclei such as ventral tegmental area, periaqueductal gray, and locus coeruleus (Amaral & Cowan, 1980; Insausti & Amaral, 2012). Furthermore, the proportion of endocrine receptor expression in the (mouse) hippocampus exceeds anything that has been observed in all comparable brain regions with the exception of the hypothalamus (Lathe et al., 2020). These findings demonstrate a clear link between hippocampal processing and allostatic concerns; for instance, the hippocampus may be key for the integration of interoceptive information with previous experiences and exteroceptive sensory signals (Quigley et al., 2021).

It remains to be discovered to what extent, if any, functional gradients in the cerebellum and the hippocampus map onto allostatic processing in the manner proposed for the gradients of the isocortex. Although speculative, one possible hypothesis emerging from current evidence is that the isocortex, the cerebellum, and the hippocampus might integrate over the same information to modulate one another as they construct prediction, prediction error, and precision signals in the service of allostasis, owing to their extensive connectivity with one another and with the subcortical structures implicated in processing of these signals.

In sensorimotor coordination, for example, the cerebellum has been traditionally thought to estimate the sensory state of the body by anticipating the consequences of motor commands (Shadmehr et al., 2010; Sokolov et al., 2017; Wolpert et al., 1998), possibly as a means to compensate for delays in sensory feedback (Sultan et al., 2012; Tanaka et al., 2020)—for instance, as signals physically travel from fingertips, through the periphery and spinal cord, and into the cerebral cortex. Extrapolating from this evidence, we hypothesize that the cerebellum may compute sensory prediction errors to tune signals of various cortical ensembles faster than sensory prediction errors computed in the cerebral cortex. This view is consistent with the ability of granule cells (the majority of cerebellar neurons) to generate action potentials that are relatively short-lived and at much higher frequencies than cerebral cortical neurons (Sultan et al., 2012). Anatomical evidence has also identified polysynaptic interconnections between the cerebellar cortex and nonprimary sensorimotor areas of the cerebral cortex (e.g., parietal association, parahippocampal, occipitotemporal, and prefrontal cortices; Apps & Watson, 2013; Kelly & Strick, 2003; Schmahmann, 1996), further supporting a domain-general view of cerebellar influence on cortical processing.

The hippocampus is thought to generate prediction signals (Barron et al., 2020; Buzsáki & Tingley, 2018; Lisman & Redish, 2009; Pezzulo et al., 2017) and facilitate reweighting of signals in the isocortex (Kumaran et al., 2016). In particular, the hippocampus may help ensure that the subsequent prediction signals generated based on the isocortical internal model are not slaves to the statistics of the external sensory environment and instead more in line with the goals of the animal (i.e., weighted for the current and predicted conditions of the body’s internal environment; Kumaran et al., 2016). By interfacing with isocortical ensembles at many levels of the predictive hierarchy, the hippocampus too may intervene at multiple points of this hierarchy to modulate cortical signaling.

These hypotheses are consistent with the increasing consensus that the cerebellum (e.g., Guell, Gabrieli, et al., 2018; King et al., 2019) and the hippocampus (e.g., Grady, 2020) are functionally heterogeneous. The cerebellum and the hippocampus may serve domain-general functions within a predictive hierarchy in the service of efficient bodily regulation, rather than supporting particular functional domains exclusively (e.g., related to motor coordination, episodic memory, or spatial cognition). This domain-general perspective may help advance both an understanding of cerebello-isocortical (Shadmehr et al., 2010; Sokolov et al., 2017), hippocampo-isocortical (Buzsáki & Tingley, 2018; Kumaran et al., 2016), and even cerebello-hippocampal (e.g., Babayan et al., 2017; Onuki et al., 2015; Watson et al., 2019) interactions, and a computational understanding of their implications for various psychological processes. Specific computational hypotheses concerning the role of hierarchical functional gradients in predictive processing still need to be directly tested. Nonetheless, the existence of the common axes of functional organization across brain structures suggests that it may be fruitful to study the human brain organization in terms of domain-general functions in the service of allostasis.

## CONCLUSIONS

Allostasis is the brain’s predictive regulation of the body’s internal milieu (Sterling, 2012). Coordination and regulation of the body’s internal systems has been hypothesized to be a basic function of the brain, either as allostasis (Barrett, 2017; Barrett & Simmons, 2015; Hutchinson & Barrett, 2019; Khalsa et al., 2018; Kleckner et al., 2017; Owens et al., 2018; Petzschner et al., 2021; Petzschner et al., 2017; Pezzulo et al., 2015; Pezzulo et al., 2021; Schulkin & Sterling, 2019; Seth & Friston, 2016; Seth & Tsakiris, 2018; Stephan et al., 2016) or as predictive regulation of the body more generally (Ainley et al., 2016; Allen & Friston, 2018; Allen et al., 2019; Seth, 2013; Seth et al., 2012; Smith et al., 2017), and therefore may be a basic feature of the mind. The brain’s modeling of the sensory consequences of allostasis, both interoceptive and exteroceptive, may translate into consciously experienced feelings of valence and arousal as basic features of consciousness (e.g., Lindquist et al., 2016; Satpute et al., 2015; for discussion, see Barrett, 2017). In this paper, we connected these hypotheses with hierarchical functional gradients that appear to organize whole-brain function (Katsumi et al., 2021; Zhang et al., 2019), which are grounded in biological structure (thus far established for the cerebral cortex). Our claim is not that allostasis is the exclusive function of the brain; rather, we suggest that behavior is always planned and executed—and mental events always emerge—in the context of regulating the body’s internal systems, and the entire brain is engaged in this regulatory function at the same time as it governs behavior and mental activity.

Although speculative, one final intriguing hypothesis emerging from this view is that all psychological phenomena (e.g., cognition, emotion, and perception) may be whole-brain phenomena with allostatic features, rather than separate states arising from unique computations that are localized to specific regions. This idea is consistent with a growing body of anatomical and functional evidence. For example, as mentioned above, exteroceptive sensory processing is statistically associated with processing of bodily signals. Primary motor cortex contains visceromotor maps (Levinthal & Strick, 2012, 2020), suggesting intimate integration of skeletomotor and visceromotor functions. The anterior cingulate cortex (visceromotor cortex) sends direct projections to neurons in V1 (Zhang et al., 2014), which may carry top-down prediction signals (Leinweber et al., 2017). Indeed, a substantial fraction of activity in the visual cortex does not depend on incoming visual input (Keck et al., 2013; Muckli et al., 2015), and the majority of synapses in V1 originate from top-down sources (Sillito & Jones, 2002). Such evidence runs counter to traditional assumptions that psychological functions can be uniquely localized to specific brain regions or networks and is consistent with the hypothesis of a domain-general computational architecture of the brain (e.g., see Barrett, 2017, for how this approach is applied to understanding the nature of emotions). This “whole-brain” view is increasingly gaining empirical support in human neuroimaging studies that are designed to be sensitive to such observations (e.g., Gonzalez-Castillo et al., 2015; Gonzalez-Castillo et al., 2012; Liang et al., 2013) and in nonhuman animal research (reviewed in Kaplan & Zimmer, 2020). An allostatically oriented whole-brain framework has the potential to unify our understanding of brain, mind, and body. Our approach offers the basis for a coherent, neurobiologically inspired research program that attempts to explain how a variety of physical and mental events emerge from the same biological mechanisms. Data-driven approaches reveal low-dimensional gradients that summarize the organizational features of the brain; biology will help us interpret them.

## ACKNOWLEDGMENTS

The views, opinions, and/or findings contained in this review are those of the authors and shall not be construed as an official Department of the Army position, policy, or decision, unless so designated by other documents; nor do they necessarily reflect the views of the Elizabeth R. Koch Foundation. The authors thank Nada Kamona and Liz Cory for their assistance with figure creation.

## AUTHOR CONTRIBUTIONS

Yuta Katsumi: Investigation; Writing – original draft. Jordan E. Theriault: Writing – review & editing. Karen S. Quigley: Writing – review & editing. Lisa Feldman Barrett: Conceptualization; Funding acquisition; Investigation; Supervision; Writing – review & editing.

## FUNDING INFORMATION

Lisa Feldman Barrett, National Science Foundation, Award ID: BCS 1947972. Lisa Feldman Barrett, the US Army Research Institute for the Behavioral and Social Sciences, Award ID: W911NF-16-1-019. Lisa Feldman Barrett, Elizabeth R. Koch Foundation, Unlikely Collaborators Fund. Lisa Feldman Barrett, National Institutes of Health (https://dx.doi.org/10.13039/100000002), Award ID: R01 MH113234. Lisa Feldman Barrett, National Institutes of Health (https://dx.doi.org/10.13039/100000002), Award ID: R01 MH109464. Lisa Feldman Barrett, National Institutes of Health (https://dx.doi.org/10.13039/100000002), Award ID: U01 CA193632.

## TECHNICAL TERMS

Allostasis: The process of activating physiological systems (such as hormonal, autonomic, or immune systems) with the aim of returning the body to homeostasis.
Allocortex: Part of the cerebral cortex with the simplest structure (two or three layers). It comprises the hippocampus and the primary olfactory cortex (part of the cerebral cortex that receives the projection from the olfactory bulb).
Limbic cortices: Part of the isocortex with agranular or dysgranular structure. They are sometimes referred to as periallocortex (agranular) and proisocortex (dysgranular) cortex.
Visceromotor cortices: Agranular isocortical regions that modulate the regulation of the autonomic nervous system as well as of the hormonal and immune systems.
Interoception: The perception and integration of autonomic, hormonal, visceral, and immunological homeostatic signals that collectively describe the physiological state of the body.
Agranular cortex: An isocortical region with neurons that configure into a relatively undifferentiated superficial layer (corresponding to layers II and III) and lacking a fully differentiated layer IV.
Granular cortex: An isocortical region with six differentiated layers, including a well-defined layer IV that contains many stellate granule cells receiving thalamocortical inputs.
Dysgranular cortex: Part of the isocortex with a rudimentary layer IV.
(Functional) connectivity gradients: Low-dimensional representations of similarity in connectivity profiles across a set of data points (e.g., voxels, surface vertices, or regions of interest).
